# Endothelial Cells Promote Osteogenesis by Establishing a Functional and Metabolic Coupling With Human Mesenchymal Stem Cells

**DOI:** 10.3389/fphys.2021.813547

**Published:** 2022-01-11

**Authors:** Sara Petrillo, Tullio Genova, Giorgia Chinigò, Ilaria Roato, Giorgia Scarpellino, Joanna Kopecka, Fiorella Altruda, Emanuela Tolosano, Chiara Riganti, Federico Mussano, Luca Munaron

**Affiliations:** ^1^Molecular Biotechnology Center (MBC), Department of Molecular Biotechnology and Health Sciences, University of Turin, Turin, Italy; ^2^Department of Life Sciences and Systems Biology, University of Turin, Turin, Italy; ^3^Department of Surgical Sciences, C.I.R. Dental School, University of Turin, Turin, Italy; ^4^Department of Oncology, University of Turin, Turin, Italy

**Keywords:** HMEC, endothelial cell (EC), osteogenesis, bone regeneration, bone biology, metabolism, mesenchymal stem cell (MSC)

## Abstract

Bone formation involves a complex crosstalk between endothelial cells (EC) and osteodifferentiating stem cells. This functional interplay is greatly mediated by the paracrine and autocrine action of soluble factors released at the vasculature-bone interface. This study elucidates the molecular and functional responses triggered by this intimate interaction. In this study, we showed that human dermal microvascular endothelial cells (HMEC) induced the expression of pro-angiogenic factors in stem cells from human exfoliated deciduous teeth (SHED) and sustain their osteo-differentiation at the same time. In contrast, osteodifferentiating SHED increased EC recruitment and promoted the formation of complex vascular networks. Moreover, HMEC enhanced anaerobic glycolysis in proliferating SHED without compromising their ability to undergo the oxidative metabolic shift required for adequate osteo-differentiation. Taken together, these findings provide novel insights into the molecular mechanism underlying the synergistic cooperation between EC and stem cells during bone tissue renewal.

## Introduction

In the last two decades, countless studies have focused on the osteogenesis-angiogenesis binomial for its pivotal role in bone regeneration. In fact, bone tissue renewal requires both osteogenic and angiogenic processes to ensure the adequate bone formation and tissue regeneration. Therefore, great efforts have been made to optimize the features of the two main actors involved in bone regenerative approaches, i.e., cells and scaffolds. The formers are responsible for the high regenerative potential and self-renewing properties of bones, whereas scaffolds supply a three-dimensional structure suitable for housing the cells, thus supporting them mechanically and functionally (Matassi et al., 2011; Genova et al., 2019). One of the biggest challenges in tissue engineering remains to obtain an adequately vascularized scaffold to sustain and optimize bone regeneration. In fact, it has been largely demonstrated that the efficiency of the graft, in terms not only of bone development but also of long-term graft survival, is strongly determined by the ability of the scaffold to guarantee the development of vascular networks (Mussano et al., 2018; Cipriano et al., 2020; Diomede et al., 2020). For this purpose, it is desirable to have a porous and biocompatible scaffold material with remarkable osteoinductive properties as well as a high degree of interconnectivity of the pores, which allows endothelial cell (EC) migration and proliferation and thus the development of a functional vascular network (Chazono et al., 2008; Chantarapanich et al., 2012; Sekiya et al., 2013; Diomede et al., 2018a). In addition, many studies highlighted the great potential of coculturing EC and osteogenic cells to enhance vessel formation as well as bone repair at once (Clarkin et al., 2008; Liu et al., 2013; Herzog et al., 2014; Jiang et al., 2018; Genova et al., 2019). Thus, the use of co-culture systems draws much attention in bone tissue engineering (Grellier et al., 2009), investigating the intimate connection and the virtuous circuit established between angiogenic and osteogenic pathways in bone tissue renewal. In this view, a deeper knowledge of the mechanisms underlying such functional relationship seems crucial for approaches aimed at bone engineering improvement.

The oral cavity can be considered a promising source of human mesenchymal stem cells (MSC) to be enrolled in bone tissue engineering approaches. The major advantages associated with oral MSC, in addition to showing common features to MSCs deriving from the bone marrow, include their simple obtaining, isolation, and manipulation (Trubiani et al., 2019; Roato et al., 2021). Moreover, MSCs deriving from the oral cavity display a high-expansion capability, great differentiating potential, and marked immunomodulatory activity exerted through extracellular vesicles (EV) and paracrine signals (Diomede et al., 2018b; Pizzicannella et al., 2019a,b). Finally, several data revealed a good potential of oral MSC in inducing angiogenesis through a promising endothelial commitment (Pizzicannella et al., 2019b), thus leading to enhanced *in vitro* and *in vivo* bone formation (Bakhtiar et al., 2018; Xuan et al., 2018; Guo et al., 2020). In addition to their high osteo-differentiation capacity, the central role played by MSC in tissue renewal, repair, and healing is related to their paracrine functions, i.e., the release of growth factors and other biologically active molecules. Nevertheless, this crosstalk is bidirectional, and many paracrine signals are also produced by EC, including growth factors, hormones, and other promoters of bone cell growth, differentiation, and regeneration (Guillotin et al., 2004; Zhang et al., 2012; Leszczynska et al., 2013). Well-established is the role played by the vascular endothelial growth factor-A (VEGF-A). In fact, it is known that, beyond enhancing EC migration, proliferation, and vessel permeability (van Gastel et al., 2012; Cai et al., 2017), VEFG-A indirectly stimulates MSC osteo-differentiation (Clark et al., 2015; Diomede et al., 2020). Secreted factors have also been involved in the immunomodulatory responses, which importantly minimize the risk of autoimmune rejection (Moshaverinia et al., 2014; Diomede et al., 2020; Gomez-Salazar et al., 2020). Furthermore, an active role in osteogenic-angiogenic coupling has been recently attributed to micro-RNAs (miRNA) (Fröhlich, 2019). Finally, the exchange of metabolites at the vasculature-bone interface appears fundamental in bone tissue formation. In fact, growing evidence shows that metabolism drives expansion and osteo-differentiation of MSC (Chen et al., 2008, 2016; Hsu et al., 2013; Liu and Ma, 2015; Li et al., 2017).

A deeper understanding of the paracrine signaling underlying the dense endothelium-stem cell crosstalk could provide new knowledge useful for the development of improved therapeutic strategies in bone regeneration and tissue engineering. Interestingly, the delivery of specific growth factors and signaling molecules through extracellular vesicles could implement MSC-based therapy providing some benefits, such as avoiding ethical concerns and limitations due to the administration of living cells. Moreover, studies on MSC metabolism may offer new insights in optimizing the translation of MSC-based therapy to clinical application, to enhance its therapeutic effects (Liu and Ma, 2015).

In this study, we aimed to deepen the functional relationship between human dermal microvascular endothelial cells (HMEC) and stem cells from human exfoliated deciduous teeth (SHED), one of the most promising oral MSCs thanks to its ability to preserve an even greater growth potential as compared with MSC from bone marrow (BM-MSC) (Pizzicannella et al., 2018). More specifically, we first evaluated the osteogenic and angiogenic potential of SHED. Then, we elucidated the metabolic cooperation underlying the functional crosstalk between SHED and HMEC from the sustained aerobic glycolytic state, which supports MSC expansion (Lunt and Vander Heiden, 2011), to the oxidative metabolic rewiring needed to fulfill the high energy demand of osteo-differentiating stem cells (Chen et al., 2008).

## Materials and Methods

### Cell Culture

Two cell types were used, namely, HMEC and SHED. HMEC were purchased from Lonza (Lonza, Switzerland) and were grown in complete EndoGRO-MV (Millipore, Italy) supplemented with 50 μg/ml gentamicin (Cambrex) (Genova et al., 2019). HMEC were used up to passage six. SHED was cultured following their harvest until the fourth passage. SHED was obtained and manipulated as described elsewhere (Mussano et al., 2018). This study was conducted following the protocols approved (in October 2012) by the Ethics Committee of the CIR-Dental School of the University of Turin (harvest of deciduous teeth, protocol number CIR20121022). SHED was extracted from integer exfoliated deciduous teeth that were collected from 10 children (9.2 ± 2.2 years) undergoing tooth extraction at the CIR-Dental School. Written informed consent was always obtained from donors.

### Flow Cytometry Analysis of Mesenchymal Stem Cell Phenotype

Cell surface markers of SHED were analyzed by flow cytometry, identifying MSC as positive for the mesenchymal markers CD105, CD44, CD73, and CD90 and negative for CD45 expression (Dominici et al., 2006). Standard labeling protocol was performed with the following fluorochrome-conjugated antibodies and isotypic controls: human CD105 PE (Thermo Fisher Scientific), CD73 FITC, CD90 PerCP (Biolegend), CD44 FITC, CD45 PerCP, IgG1 PE, and IgG2a PerCP (Miltenyi Biotech), and IgG1 FITC-conjugated (Immunostep). As a further control, unstained cells were also examined. Data were acquired using MACsQuant 10 Cytometer and analyzed through MACsQuantify software (Miltenyi Biotech).

### Osteogenic Cell Differentiation

To obtain osteogenic differentiation, 10 × 10^4^/well SHED were cultured in a six-well plate in osteo-differentiating medium (OM) for 7 days by supplementing the normal growth medium with 10 mM β-glycerophosphate, 50 μg/ml ascorbic acid, and 0.02 mg/ml dexamethasone. Prior to the experiment, dexamethasone was removed to avoid any inhibitor effect on EC as reported in the literature (Mussano et al., 2017).

### Proliferation Assay

Stem cells from human exfoliated deciduous teeth were seeded at a density of 1 × 10^3^ cells/well in 96-well culture dishes and maintained in basal growth medium (GM) or OM, respectively. The proliferation was assessed using an automated cell counter (Thermo Fisher Scientific) at 1, 3, and 7 days of culture.

### RNA Extraction and Real-Time PCR Analysis

Stem cells from human exfoliated deciduous teeth, maintained in OM or GM, were seeded on a 6-well plate and cocultured or not with HMEC seeded on 6-well 0.4-μm pore polycarbonate membrane inserts. RNA extraction and quantitative real-time PCR (qRT-PCR) analyses were performed as previously described (Petrillo et al., 2018). In brief, the total RNA was extracted using the PureLink RNA Mini Kit (Thermo Fisher Scientific, Waltham, MA, United States), and 0.5–1 μg of total RNA were transcribed into complementary DNA (cDNA) using the High-Capacity cDNA Reverse Transcription Kit (Thermo Fisher Scientific, Waltham, MA, United States). qRT-PCR was performed using gene-specific TaqMan™ Gene Expression Assays (Thermo Fisher Scientific Waltham, MA, United States). The following primers were used: VEGFA: Fw-ctacctccaccatgccaagt, Rev-ccatgaacttcaccacttcgt; ANGPT1: Fw-gacagatgttgagacccaggta, Rev-tctctagcttgtaggtggataatgaa; PDGFb: Fw-tgatctccaacgcctgct, Rev-tcatgttcaggtccaactcg; HGF: Fw-gattggatcaggaccatgtga, Rev-ccattctcattttatgttgctca; TGFb: Fw-actactacgccaaggaggtcac, Rev-tgcttgaacttgtcatagatttcg; and BMP2: Fw-gactgcggtctcctaaaggtc, Rev-ggaagcagcaacgctagaag. qRT-PCR was performed on a 7900HT Fast or QuantStudio™ 6 Flex Real-Time PCR System (Thermo Fisher Scientific, Waltham, MA, United States), and the analyses were performed using RQ Manager or QuantStudio Real-Time PCR software. Transcript abundance, normalized to 18s messenger ribonucleic acid (mRNA) expression, is expressed as a fold change over a calibrator sample.

### Chemotaxis Assay

Human dermal microvascular endothelial cells were seeded into transwell inserts with 8 μm pore (0.5 × 10^4^/transwell) and maintained in EndoGRO-MV (Millipore, Italy). The following day, transwells with HMEC were put into a 24-well plate (bottom chamber) with SHED maintained in growth medium (SHED-GM) or SHED maintained in osteo-differentiating medium (SHED-OM). After 4 h (h), transwell inserts were fixed with 4% paraformaldehyde and stained with DAPI. Non-migrated cells were removed using a cotton swab. Total migrated cells (nuclei) were counted.

### Migration Assay

Cell motility was investigated as the migration of cells into a wound introduced in a confluent monolayer. HMEC were grown to confluence on 24-well culture plates. A “wound” was made by scraping the middle of the cell monolayer with a P10 pipette tip. Then, HMEC were put in coculture with SHED-GM or SHED-OM using 24-well 0.4 μm pore polycarbonate membrane inserts. Experiments were performed using a Nikon Eclipse Ti-E microscope with a 4 × objective. Cells were kept at 37°C and 5% CO_2_ for all experiments, and the acquisition was obtained using Metamorph software (Molecular Devices, Sunnyvale, California, United States) (Avanzato et al., 2016; Canullo et al., 2016, 2017). Cell motility into a wound was measured after 8 h using Metamorph software and was expressed as the percentage of cell migration (Fiorio Pla et al., 2010, 2014; Basilico et al., 2015). At least three fields for each condition were analyzed in each independent experiment. At least three independent experiments were performed for each experimental condition.

### *In vitro* Angiogenesis Assay

*In vitro* formation of capillary-like structures was performed on growth factor-reduced Matrigel (Corning, United States) in 24-well plates. HMEC (3.5 × 10^4^ cells/well) were seeded on the Matrigel. Then, HMEC were put in a coculture with SHED-GM or SHED-OM using 24-well 0.4-μm pore polycarbonate membrane inserts. Cell organization in Matrigel was acquired after 8 h using a Nikon Eclipse Ti E microscope using a Nikon Plan 10 × /0.10 objective. At least three independent experiments were performed for each experimental condition (Genova et al., 2017). Using the angiogenesis analyzer tool of ImageJ developed by Gilles Carpentier, several parameters were analyzed: (1) nodes are pixels with three neighbors represented as a circular dot; (2) junctions correspond to nodes or groups of fusing nodes; (3) segments are elements delimited by two junctions; (4) isolated elements are binary lines that are not branched; (5) master segments consist in pieces of tree delimited by two junctions none exclusively implicated with one branch, called master junctions; and (6) master junctions are junctions linking at least three master segments. Optionally, two close master junctions can be fused into a unique master junction. With the term “tree,” we identified the complex structure made of nodes and segments that EC form *in vitro* during the experiment.

### Activity of Glycolytic Enzymes and Lactate Dehydrogenase

Human dermal microvascular endothelial cell-conditioned medium (CM) was collected after 72 h, diluted with GM or OM (1:2), respectively, and used to treat SHED for 24 h. SHED was washed with fresh medium, detached with trypsin/EDTA, resuspended at 1 × 10^5^ cells/ml in 0.2 ml of 100 mM TRIS 10 mM/EDTA I mM (pH 7.4), and sonicated on ice with two 10 s bursts. Enzymatic activities were measured on 10 μl cell lysates, incubated for 5 min at 37°C. The protein content was measured using the BCA1 Kit (Sigma, St. Louis, MO, United States). The activity of phosphofructokinase-1 (PFK1) assay was measured spectrophotometrically as reported in the study by Sharma (2011). The activities of glyceraldehyde 3-phosphate dehydrogenase (GAPDH), enolase (ENO), pyruvate kinase (PK), and lactate dehydrogenase (LDH) were measured spectrophotometrically according to the study by Riganti (Riganti et al., 2002; Capello et al., 2016). For GAPDH, cell lysate was incubated with 5 mM 3-phosphoglyceric acid, 1 U phosphoglycerate 3-kinase, 5 mM ATP, and 2.5 mM NADH. For enolase, cell lysate was incubated with 10 mM MgCl_2_, 100 mM KCl, 1 mM 2-phosphoglyceric acid, 0.4 mM ADP, 6.8 U/ml PK, 9.9 U/ml LDH, and 0.2 mM NADH. For all assays of glycolytic enzymes, the activities were monitored measuring the absorbance variation at 340 nm using a Synergy HTX 96-well microplate reader (Bio-Tek Instruments). The kinetics was linear throughout the measurement.

### Activity of Mitochondrial Electron Transport Chain Complexes I–IV

Human dermal microvascular endothelial cell CM was collected after 72 h, diluted with GM or OM (1:2), respectively, and used to treat SHED for 24 h. According to Wibom et al. (2002), SHED were washed twice in ice-cold 0.1 M phosphate-buffered saline (PBS), then lysed in 0.5 ml buffer A (50 mmol/L Tris, 100 mmol/L KCl, 5 mmol/L MgCl_2_, 1.8 mmol/L ATP, 1 mmol/L EDTA, pH 7.2), supplemented with protease inhibitor cocktail III [100 mmol/L AEBSF, 80 mmol/L aprotinin, 5 mmol/L bestatin, 1.5 mmol/L E-64, 2 mmol/L leupeptin, and 1 mmol/L pepstatin] (Merck, Darmstadt, Germany), 1 mmol/L phenylmethylsulfonyl fluoride (PMSF), and 250 mmol/L NaF. Samples were clarified by centrifuging at 650 × *g* for 3 min at 4°C, and the supernatant was collected and centrifuged at 13,000 × *g* for 5 min at 4°C. The new supernatant was discarded, and the pellet containing mitochondria was washed in 0.5 ml buffer A and resuspended in 0.25 ml buffer B (250 mmol/L sucrose, 15 μmol/L K_2_HPO_4_, 2 mmol/L MgCl_2_, 0.5 mmol/L EDTA, and 5% w/v bovine serum albumin). A 100 μl aliquot was sonicated and used for the measurement of protein content. The remaining not-sonicated part was used to measure the electron transport chain (ETC) complex I–IV activities according to Wibom et al. (2002). Results were expressed as nmol NAD+ /min/mg mitochondrial protein for complex I, nmol cyt c reduced/min/mg mitochondrial protein for complexes II–III, and nmol cyt c oxidized/min/mg mitochondrial protein for complex IV.

### ATP Levels in Mitochondria

Human dermal microvascular endothelial cell CM was collected after 72 h, diluted with GM or OM (1:2), and used to treat SHED for 24 h. The ATP levels in SHED mitochondrial extracts were measured using the ATP Bioluminescent Assay Kit (Sigma-Aldrich, St. Louis, MO, United States). ATP was quantified as relative light units (RLU) and converted into nmol ATP/mg mitochondrial proteins, according to the calibration curve previously set.

### Statistical Analysis

Data were analyzed using GraphPad Prism6 (GraphPad Software, Inc., La Jolla, CA, United States). Each experiment was repeated at least three times. Statistical analysis was performed by using ordinary one-way ANOVA with Tukey’s multiple comparisons test, two-way ANOVA with Bonferroni’s multiple comparisons test or unpaired non-parametric Mann-Whitney *U* test. A *p*-value of <0.05 was considered significant.

## Results

### Osteo-Differentiation and Characterization of Stem Cells From Human Exfoliated Deciduous Teeth

**FIGURE 1:**
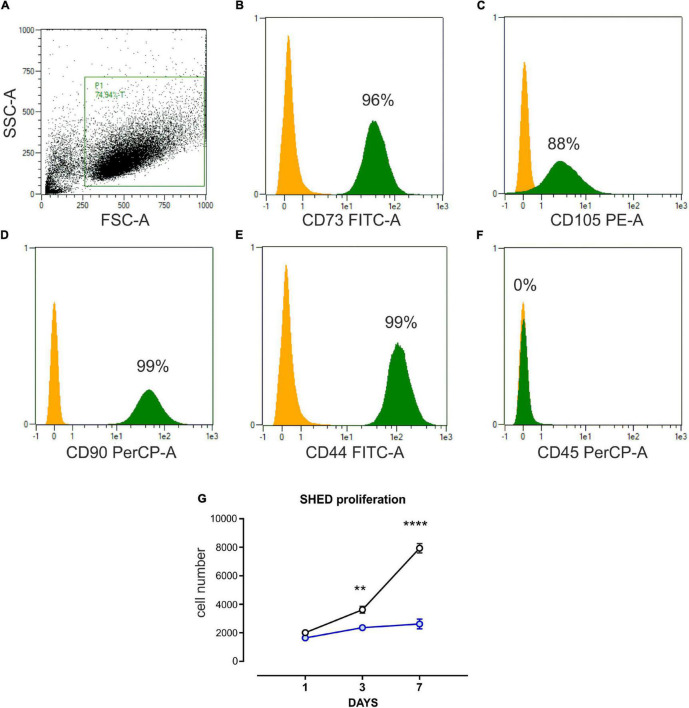
Characterization of SHED. **(A–F)** Flow cytometer analysis performed on stem cells isolated from human exfoliated deciduous teeth (SHED) and cultured *in vitro.* Undifferentiated SHED showed the typical mesenchymal phenotype, expressing CD73, CD105, CD90, CD44, and negative for CD45. P1 is the gate on mesenchymal stem cell (MSC) population, obtained after two *in vitro* passages. The yellow histogram represents the negative control, while the green one is the percentage of cells expressing the marker in P1, the MSC gate. **(G)** Cell count (DAPI staining) proliferation assay performed on SHED cultured in basal growth medium (black line) and osteo-differentiating medium (blue line), respectively. Data are expressed as mean ± SEM of *n* = 6; 2-way ANOVA with Bonferroni’s multiple comparison test was performed: **p < 0.001; ****p < 0.0001.

The purity of SHED was assessed through flow cytometry analysis after two *in vitro* cell culture passages. Consistent with their mesenchymal origin, SHED expressed CD105, CD44, CD73, and CD90 while resulting negative for CD45 (Figures 1A–F; Dominici et al., 2006). To induce osteogenic differentiation, SHED were maintained in OM for 7 days. A proliferation assay was performed at multiple time points to keep track of the osteogenic differentiation process. As shown in Figure 1G, the proliferative rate of SHED kept in OM was significantly reduced compared to SHED cultured in the presence of GM. These results indicate that SHED cultured in OM undergoes a proliferative stop while acquiring an early differentiated state.

### Endothelial Cells Induce the Expression of Pro-angiogenic Factors in Stem Cells From Human Exfoliated Deciduous Teeth and Promote Their Osteo-Differentiation

**FIGURE 2:**
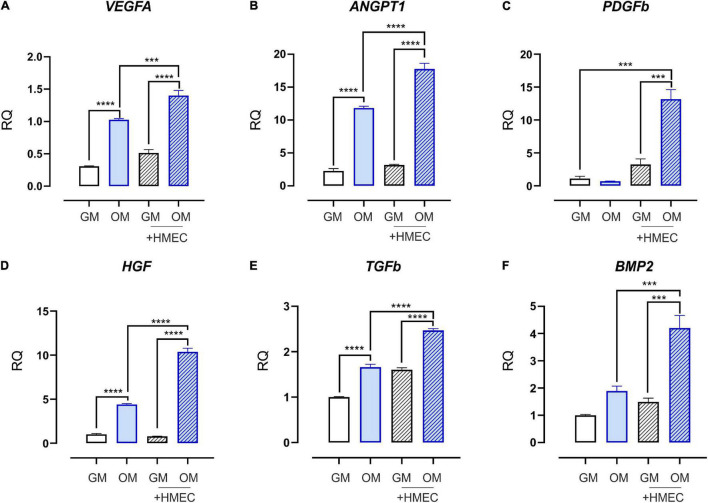
Expression profile of growth and angiogenic factors in SHED cocultured with HMEC. qRT-PCR analysis performed on SHED under basal conditions (without EC) or after 4 h of coculture with HMEC. Undifferentiated (white bars) and osteodifferentiating (blue bars) SHED is shown. Data represent mean ± SEM; *n* = 3. ***p < 0.001; ****p < 0.0001. Statistical analysis: ordinary one-way ANOVA with Tukey’s multiple comparisons test. **(A)** *VEGFA*, vascular endothelial growth factor A; **(B)** *ANGPT1*, angiopoietin 1; **(C)** *PDGFb*, platelet-derived growth factor b; **(D)** *HGFb*, hepatocyte growth factor b; **(E)** *TGFb*, transforming growth factor b; **(F)** *BMP2*, bone morphogenetic protein 2.

To elucidate the functional interplay resulting from cell-cell communication without direct physical interaction, human dermal microvascular EC (HMEC) were cocultured with SHED-GM or SHED-OM. Then, the expression of some pro-angiogenic factors was evaluated. Importantly, the OM condition was sufficient to induce the expression of pro-angiogenic factors vascular endothelial growth factor A (VEGFA, Figure 2A), angiopoietin 1 (ANGPT1, Figure 2B), hepatocyte growth factor (HGF, Figure 2D), and transforming growth factor-beta (TGFb, Figure 2E). These data suggest that osteodifferentiating SHED stimulates EC recruitment to ensure proper vascularization during osteogenesis. Moreover, the presence of EC further enhanced this process, by promoting an even stronger induction of pro-angiogenic factors in SHED cultured in OM (Figures 2A–E). These findings demonstrate the ability of EC to stimulate the release of pro-angiogenic factors by osteodifferentiating stem cells to promote angiogenesis and further recruitment of EC. Importantly, the expression of the main marker of osteo-differentiation, i.e., bone morphogenetic protein 2 (BMP2), was strongly increased in osteodifferentiating SHED cocultured with HMEC as compared to osteodifferentiating SHED alone, showing that EC also promotes SHED osteo-differentiation (Figure 2F). Taken together, these data reveal a virtuous loop in which EC and SHED are functionally coupled in osteogenesis and angiogenesis regulation.

### Osteo-Differentiating Stem Cells From Human Exfoliated Deciduous Teeth Promote Recruitment, Migration, and Angiogenesis of Human Microvascular Endothelium

**FIGURE 3:**
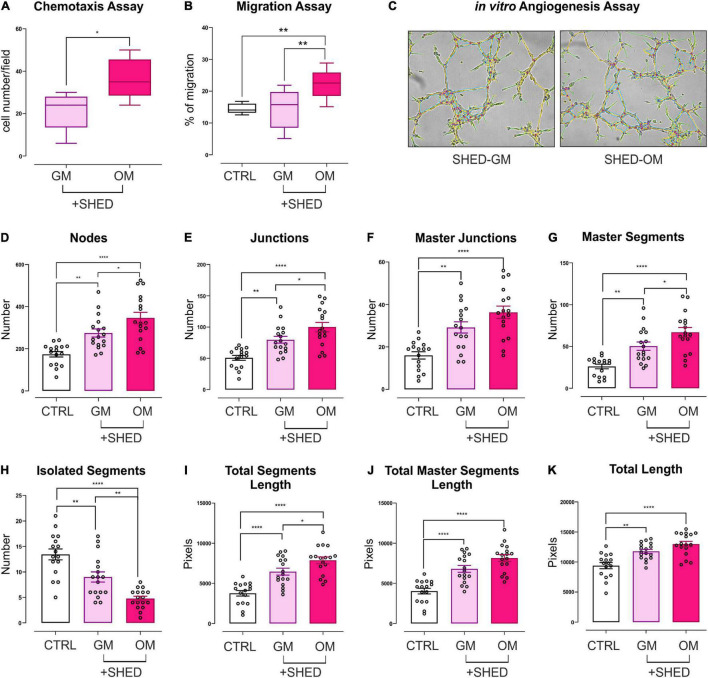
Evaluation of the angiogenic potential of HMEC conditioned by SHED. **(A)** Chemotaxis assay performed on HMEC cocultured with SHED-GM or SHED-OM. Data represent Min to Max; *n* = 5. *p < 0.05. Statistical analysis: unpaired non-parametric Mann Whitney test. **(B)** Migration assay performed on HMEC alone or cocultured with SHED-GM or SHED-OM. Data represent Min to Max; *n* = 12. **p < 0.01. Statistical analysis: ordinary one-way ANOVA with Tukey’s multiple comparisons test. **(C)** Representative pictures of *in vitro* tubulogenesis assay performed on HMEC cocultured with SHED-GM or SHED-OM. **(D–K)** The formation of capillary-like structures was performed on Matrigel coating and analyzed after 8 h using ImageJ’s Angiogenesis Analyzer tool. Data represent MEAN ± SEM; *n* = 17. *p < 0.05; **p < 0.01; ****p < 0.0001. Statistical analysis: ordinary one-way ANOVA with Tukey’s multiple comparisons test.

To elucidate the biological effects of osteo-differentiating stem cells on EC, HMEC functions were evaluated in coculture with SHED-GM or SHED-OM. First, the ability of SHED to recruit EC was tested by performing a chemotaxis-based assay. Osteodifferentiating SHED showed a significantly increased ability to attract EC as compared to undifferentiated SHED (Figure 3A). Vascular network formation requires EC migration as the first step. For this reason, a migration assay was performed on HMEC cultured alone or in the presence of osteo-differentiating and undifferentiated SHED. As reported in Figure 3B, osteodifferentiating SHED showed a higher ability to promote EC migration compared to undifferentiated ones. The presence of a mature and functional vascular network is essential to provide nutrients and oxygen to the forming bone tissue. To deeply investigate how SHED modulate angiogenesis, *in vitro* tubulogenesis assay was performed with HMEC cultured on Matrigel in the presence of osteo-differentiating or undifferentiated SHED (Figure 3C). A complete characterization of the complexity of the newly formed capillary network was performed (Figures 3D–K). The number of nodes, junctions, and segments represent the complexity of the interconnections inside the network (Figures 3D–H). Total length, total segments length, and total master segments length are indicators of the capillary network complexity and maturation in space (Figures 3I–K). Taken together, these parameters indicated that HMEC cocultured with osteodifferentiating SHED gave rise to a capillary network characterized by higher complexity as compared to HMEC cocultured with undifferentiated SHED. In conclusion, these data demonstrate the osteo-differentiating stem cells trigger a strong pro-angiogenic response in EC, aimed at favoring a proper vascularization of the forming bone tissue.

### Endothelial Cells Enhance Anaerobic Glycolysis and Reduce Oxidative Metabolism in Undifferentiated Stem Cells From Human Exfoliated Deciduous Teeth

**FIGURE 4:**
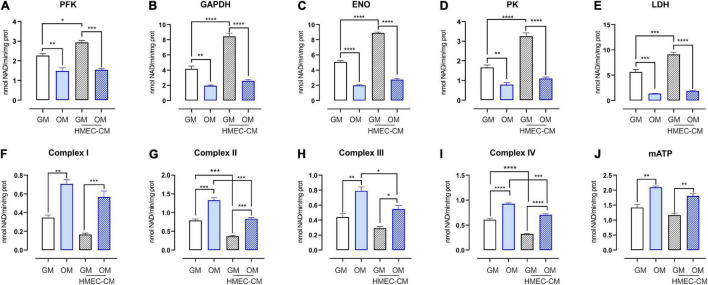
Metabolic profile of SHED treated with CM from HMEC. **(A–D)** Activities of glycolytic enzymes in SHED-GM or SHED-OM treated or not with HMEC derived CM. Data represent means ± SEM, *n* = 3. For statistical analyses, ordinary one-way ANOVA with Bonferroni’s multiple comparison test was used. *p < 0.05; **p < 0.01, ***p < 0.001, ****p < 0.0001. **(E)** Activity of lactate dehydrogenase (LDH) in SHED-GM or SHED-OM treated or not with HMEC derived CM. Data represent means ± SEM, *n* = 3. For statistical analyses, ordinary one-way ANOVA with Bonferroni’s multiple comparison test was used. ***p < 0.001, ****p < 0.0001. **(F–I)** Activities of mitochondrial ETC complexes I–IV in SHED-GM or SHED-OM treated or not with HMEC derived CM. Data are expressed as nmol NAD+/min/mg of mitochondrial protein for complex I, nmol reduced cytochrome c/min/mg of mitochondrial protein for complexes II–III, and nmol oxidized cyto-chrome c/min/mg of mitochondrial protein for complex IV. Data represent means ± SEM, *n* = 3. For statistical analyses, ordinary one-way ANOVA with Bonferroni’s multiple comparison test was used. *p < 0.05; **p < 0.01; ***p < 0.001; ****p < 0.0001. **(J)** Mitochondrial ATP levels measured using a bioluminescent assay kit in SHED-GM or SHED-OM treated or not with HMEC derived CM. Results are expressed as nmol/mg of mitochondrial proteins. Data represent means ± SEM, *n* = 3. For statistical analyses, ordinary one-way ANOVA with Bonferroni’s multiple comparison test was used. **p < 0.01. PFK, phosphofructokinase-1; GAPDH, glyceraldehyde 3-phosphate dehydrogenase; ENO, enolase; PK, pyruvate kinase; LDH, lactate dehydrogenase; mATP, mitochondrial ATP.

Endothelial cells contribute to bone development not only by forming new blood vessels, but also by providing membrane-bound and secreted elements at the vasculature-bone interface, thus supporting osteoprogenitors self-renewing as well as their differentiation into osteoblasts. The profile of secreted elements provided by the angiocrine endothelium also includes metabolites that ensure a complex and bi-directional metabolic coupling among cells. To elucidate how EC could affect the metabolic profile of SHED, conditioned-medium experiments were performed. In particular, undifferentiated and osteo-differentiating SHED was treated for 72 h with CM derived from HMEC. Later, glycolysis and mitochondrial oxidative phosphorylation (OXPHOS) were evaluated. As expected, osteodifferentiating SHED displayed a strong induction of the activity of mitochondrial electron transport chain (ETC) complexes, which in turn promoted higher adenosine triphosphate (ATP) production (Figure 4). Conversely, the activity of key glycolytic enzymes (i.e., phosphofructokinase, glyceraldehyde-3-phosphate dehydrogenase, enolase, and pyruvate kinase) and lactate dehydrogenase was significantly reduced in osteo-differentiating SHED (Figure 4). Notably, EC-derived CM significantly enhanced the glycolytic rate of proliferating SHED while lowering mitochondrial respiration (Figure 4). Nevertheless, HMEC-conditioned SHED was still able to become oxidative when cultured in OM (Figure 4). Taken together, these data demonstrate that EC improves anaerobic glycolysis in undifferentiated MSC without compromising their ability to undergo the oxidative metabolic switch required to osteo-differentiate.

## Discussion

The crosstalk between EC and osteoblasts is one of the key cellular interactions that underlie bone formation. This communication can occur by direct cell-cell contact or through the exchange of soluble factors with paracrine and autocrine actions. Moreover, soluble elements released at the osteogenic-angiogenic interface can trigger functional responses in both cell types, thus supporting angiogenesis on one side and osteogenesis on the other side. Consistently, gene expression analysis on coculture experiments showed that EC induces the release of pro-angiogenic factors, thus establishing a positive loop promoting further EC recruitment. Osteodifferentiating SHED enhanced chemoattraction and migration in neighbor EC and promoted their ability to organize into a functional and mature vascular network. Moreover, an increased expression of osteogenic marker BMP2 was found in osteo-differentiated SHED cultured with EC as compared with osteo-differentiated SHED alone. Taken together, these findings suggest that EC not only promote the release of proangiogenic factors within the forming bone tissue but also sustain stem cell osteo-differentiation. In the translation of MSC-based therapy to clinical application, MSC metabolic profile, as well as the metabolic rewiring that underlies the differentiation process, has gained growing attention in the last decades (Chen et al., 2008; Liu and Ma, 2015). Recent evidence shows that undifferentiated MSC has low levels of mitochondrial activity and depends on glycolysis for energy production (Chen et al., 2008; Liu and Ma, 2015; Li et al., 2017). Conversely, osteogenic differentiation is associated with a significant enhancement of mitochondrial oxidative metabolism in response to a higher energy demand whereas the glycolic activity is strongly reduced (Chen et al., 2008; Liu and Ma, 2015; Li et al., 2017). In this study, we found that the EC-derived CM made proliferating SHED more glycolytic than untreated proliferating SHED. In regenerative medicine, hMSCs face a hypoxic environment at the transplantation site within bone fracture usually characterized by damaged blood vessels (Brighton and Krebs, 1972; Raheja et al., 2010). Limited oxygen perfusion is also found in three-dimensional culture scaffolds (Buizer et al., 2018). Based on this evidence, we hypothesized that strategies aimed to make the grafted cells more dependent on anaerobic glycolysis could enhance their proliferation and expansion potential in the hypoxic environment. In this study, we found that endothelium increases glycolytic metabolism in MSC, thus further reducing their dependence on aerobic mitochondrial metabolism. This metabolic rewiring could help MSC to expand under hypoxic conditions as on transplantation without compromising their ability to undergo the oxidative metabolic switch required to osteo-differentiate. This latter aspect acquires particular relevance since attenuation of the oxidative metabolic switch dampers the osteogenic differentiation capability of MSC (Hsu et al., 2013). A better understanding of the contribution of energetic metabolism in directing MSC expansion and commitment could enhance their therapeutic potential in tissue engineering and regenerative medicine (Liu and Ma, 2015). This study meets this challenge and lays the groundworks for future studies in this direction.

## Data Availability Statement

The raw data supporting the conclusions of this article will be made available by the authors, without undue reservation.

## Author Contributions

SP, TG, LM, FM, CR, ET, and FA contributed to conception and design of the study. SP, TG, GC, GS, IR, and JK performed the experiments and analyzed the results. SP and GC wrote the first draft of the manuscript. IR, CR, LM, FM, and TG wrote sections of the manuscript. All authors contributed to manuscript revision, read, and approved the submitted version.

## Conflict of Interest

The authors declare that the research was conducted in the absence of any commercial or financial relationships that could be construed as a potential conflict of interest.

## Publisher’s Note

All claims expressed in this article are solely those of the authors and do not necessarily represent those of their affiliated organizations, or those of the publisher, the editors and the reviewers. Any product that may be evaluated in this article, or claim that may be made by its manufacturer, is not guaranteed or endorsed by the publisher.
